# A Remarkable Case

**Published:** 1896-05

**Authors:** 


### A REMARKABLE CASE.

DR. PRÖLL.

(Translated by A. McNeil, M. D., San Francisco, Cal., from *Archiv für Homœopathie.*)

In 1858 a lady of the nobility came to consult me on account of her daughter. It embarrassed her to name the complaint, but she at length mustered up courage to tell me that a horrible odor streamed out of her daughter's mouth, so that she could scarcely go near her, and for a long time had ceased to embrace her. She was unable to discover a cause, and, the most dreadful of all, her daughter's affianced had promised to visit her in three months, and she could not blame him if he would break the engagement. She wished to communicate these things before I saw her daughter.

She now called her in, and I was most agreeably surprised to be introduced to a lovely being with the most superb form, like a Juno, who very shyly offered me her hand. Her eyes were red with weeping. She was about twenty-three years old; blonde hair, blue eyes; her exterior revealed no blemish. Her mouth, as well as her graceful although voluptuous form, was a model of neatness. Her teeth were faultless, not one missing; her tongue beautifully clean, and also her throat, and yet it cost me a great effort to approach her mouth, for a cadavorous fetor almost drove me away. And yet every function, including the sexual, was normal. The riddle was difficult to solve.

I returned to her examination and discovered something, viz.: a trace of a scrofulous taint, which was told me by her mother; that when the daughter was a little girl she had suffered from a scrofulous affection of the nose and eyes.

When she retired, that I might continue my investigation of her mental state, her mother related to me that she was extremely melancholy, and that now she has longings for solitude, is reserved, timid, and sad. I had to build my diagnosis and treatment on these materials.

I explain the disgusting odor by exosmosis through the walls of the net-work of veins in the mouth and throat, and that from the relaxation of the tissues generally and their want of tone which permits gases to escape (Carbonites of Sulphur? Sulphide of Hydrogen? Phosphide of Hydrogen?) from the impure blood (scrofulous dyscrasia) through the meshes of the venous coats (as the watery vapors from the unglazed porous vessels, the so-called alcaragas).

I then selected, based on the law of the similars, Aurum-met., particularly on account of the mental condition, and also on account of the scrofulous diathesis.

At first three times a day before eating, what would rest on the tip of a spoon, of the 5th dec. trituration, and after three days the 10th dec. dilution, a drop morning and evening.

Behold how my diagnosis and choice of remedy were confirmed by the results! In seven days the mother, radiant with joy, announced a considerable improvement; after fourteen days strikingly pure breath. After three weeks, when she received the 30th dilution, a drop once a week, perfect recovery, and after two months, the wedding. The bad odor never returned.

